# Behavior of Nonplastic Silty Soils under Cyclic Loading

**DOI:** 10.1155/2014/635763

**Published:** 2014-01-30

**Authors:** Nazile Ural, Zeki Gunduz

**Affiliations:** ^1^Department of Civil Engineering, Bilecik Şeyh Edebali University, 11210 Bilecik, Turkey; ^2^Department of Civil Engineering, Sakarya University, 54400 Adapazari, Turkey

## Abstract

The engineering behavior of nonplastic silts is more difficult to characterize than is the behavior of clay or sand. Especially, behavior of silty soils is important in view of the seismicity of several regions of alluvial deposits in the world, such as the United States, China, and Turkey. In several hazards substantial ground deformation, reduced bearing capacity, and liquefaction of silty soils have been attributed to excess pore pressure generation during dynamic loading. In this paper, an experimental study of the pore water pressure generation of silty soils was conducted by cyclic triaxial tests on samples of reconstituted soils by the slurry deposition method. In all tests silty samples which have different clay percentages were studied under different cyclic stress ratios. The results have showed that in soils having clay content equal to and less than 10%, the excess pore pressure ratio buildup was quicker with an increase in different cyclic stress ratios. When fine and clay content increases, excess pore water pressure decreases constant cyclic stress ratio in nonplastic silty soils. In addition, the applicability of the used criteria for the assessment of liquefaction susceptibility of fine grained soils is examined using laboratory test results.

## 1. Introduction

Silt is different from clay and sand. Sandy silt in particular tends to dilate and decrease in pore pressure due to increasing strains during shear [1]. Many researchers have studied the static and physical behavior of silt [2–7]. In some parts of the world, silty soils are widespread. Several hazards are seen during dynamic loading including substantial ground deformation, reduced bearing capacity, and liquefaction of silty soil. The issue of the liquefaction potential of sands appeared in the literature after the 1964 Niigata and 1964 Alaskan earthquakes. The response of silt to seismic activity has been investigated extensively in recent years because of unexpectedly high rates of ground failure observed following the 1975 Haicheng earthquake, 1976 Tangshan earthquake, 1989 Loma Prieta earthquake, 1994 Northridge earthquake, 1999 Kocaeli earthquake, and 1999 Chi-Chi earthquake events [8–12]. Many authors have investigated the liquefaction behavior of silt, silt clay, sandy silt, and sandy clay [13–15].

Past research mostly on sand has reached widely differing views such as stating that increasing the silt content of sand would decrease the shear strength, while increasing the resistance to liquefaction [16–18], or that the liquefaction potential would increase until a threshold value is reached, after which the increasing fine percentages would make it more liquefiable [19]. Chang et al. [20] studied the effect of silt content on the cyclic shear resistance on sand-silt mixtures. Samples were prepared to a constant void ratio using the moist tamping method. Until silt content of about 20% was reached, liquefaction resistance decreased with increased silt content. After the silt content exceeded 20%, liquefaction resistance increased. They observed that the clean sand developed a pore water pressure ratio of *r*_*u*_ = 1 without first developing significant cyclic mobility, whereas the plastic silty soil developed cyclic mobility before a pore water pressure ratio of *r*_*u*_ = 1 was recorded. Cao and Law [21] conducted cyclic triaxial tests on nonplastic silty soil samples reconstituted by the moist tamping method. Liquefaction resistance decreased with increased silt content, until the silt content reached about 60%. It was observed that when silt content exceeded 60%, liquefaction resistance increased. Koester [22] conducted cyclic triaxial tests on different amounts of low plasticity silt to sand samples reconstituted by the moist tamping method. Silt was added to the sand until the silt content reached about 60%. Liquefaction resistance decreased with increased silt content until a silt content of about 30% was reached. After the silt content exceeded 30%, liquefaction resistance increased.

Singh [23] conducted cyclic triaxial tests on specimens prepared by the moist tamping method. He showed that sand containing 10, 20, or 30% silt by weight had less resistance to liquefaction than clean sand for a given relative density. Also, he showed that the pore water pressure buildup and cyclic mobility characteristics of nonplastic silt were similar to those commonly known for clean sand and that the pore water pressure buildup and cyclic mobility characteristics of plastic silt and plastic sand were similar. Erten and Maher [24] conducted cyclic triaxial tests to investigate the effect of fine content on pore pressure generation in sand. They showed that the pore pressure generated increased with an increase in fine content up to 30%. The addition of low plasticity silt to sand had no significant effect on the generated pore pressures, up to 60% silt content. The increase in pore water pressure was significantly reduced in samples with silt content above 60%. Das et al. [25] studied the liquefaction of silty soil using a cyclic triaxial test. They indicated that silty soil specimens were susceptible to failure by large axial deformations even though loss of initial effective confining pressure may not occur.

Atukorala et al. [26] examined laboratory cyclic triaxial and simple shear test data on silty soil. The cyclic triaxial test results indicated that the strain development and pore pressure generation characteristics of silty soil are generally different from those of sandy soil. The pore pressure and strain development in silty soil occur gradually with the increasing number of cycles, whereas in sandy soil pore pressure and strain development occur rapidly during the last few cycles of loading. Therefore, sudden or “brittle” collapse of foundation soil appears to be unlikely in silty soil subjected to seismic shaking. Sunitsakul [27] reported that the dynamic behavior of fine-grained soil or silty soil is heavily influenced by the frequency and the amplitude of ground motions. Hyde et al. [28] conducted cyclic triaxial testing on the effects of cyclic loading on reconstituted samples of low plasticity silt. They showed three schematic diagrams that explain the contractive and dilative shear behavior of both undrained monotonic and cyclic loading.

The sample used for this study was taken from the outskirts of Adapazarı city at a depth of 3-4 m and is believed to be of fluvial origin. The Adapazarı silt had been deposited recently by the floods of the Sakarya River within the past few millennia. The soil of Adapazarı was formed by deep sediment transported by the Sakarya and Mudurnu Rivers forming the km long Akova, to the south of the North Anatolian Fault. Most of Adapazarı is located over deep alluvial sediment. Komazwa et al. [29] have stated that the depth of the sediment reaches 1000 m in the center of the city. Adapazarı has suffered heavy damage as a result of frequent earthquakes in the past. In particular, during the Kocaeli earthquake of 17 August, 1999, buildings were strongly shaken in Adapazarı city. In this paper, a series of undrained cyclic triaxial tests was carried out on silty soil specimens with void ratios between 0.75 and 0.80. In addition, the effects of cyclic stress ratio (CSR = 0.15, 0.20, 0.25, and 0.375) and fine content and clay content on these pore pressures were studied. The laboratory testing procedure and experimental data are presented in the following sections.

## 2. Experimental Procedure

### 2.1. Materials

Silts are special materials. Silt can essentially be viewed as very fine sand. Although silt is mostly composed of quartz, it often behaves like clay. However, the behavior of silt is deemed to be unpredictable due to its dilative and contractive properties. Silt is usually encountered along rivers, deltas, and estuaries. Silt can travel far by water and wind due to its fine size. The silt used for testing was procured from Adapazarı city, Serdivan District, and the approximate limit of the annual floods of the Sakarya River and contains about 10% clay-sized particles. It was excavated by mechanical shovel, air-dried, and stocked in the laboratory. Samples were then prepared by mixing the soil with water about ten times of its volume and the supernatant clay was vacuumed out at different intervals to prepare samples with 4, 6, and 9 percent clay content, whereas the natural clay was added to obtain samples with more than 10% clay. Another mixture sample was obtained with the addition of clay to the first soil.

**Figure 1:**
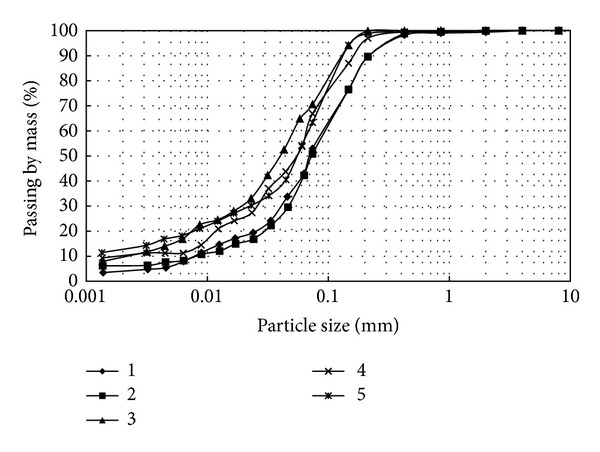
Grain size distribution of materials used.

Five different samples were prepared for dynamic testing with the same clay mineral whose basic properties are shown in Table 1. The grain size distribution as TS-1900 [30] of the silty soil is presented in Figure 1. As per the IS classification system, the quarry dust was identified as inorganic silt with the symbol ML. The liquid limit of the mixtures was determined in the BS/TS fall cone apparatus as it was discovered that measurement was not possible on samples with the percussion apparatus.

### 2.2. Sample Preparation

The degree of disturbance is most pronounced in silt, which practically prevents researchers from obtaining even slightly disturbed samples. It was therefore decided to use reconstituted samples for testing rather than using undisturbed samples with unknown degrees of disturbance. It is important to select the method of preparation for reconstituted samples which will realistically simulate natural samples. Methods such as dry pluviation, wet tamping, sedimenting, and slurry deposition have been developed for the purpose. Generally in the literature, the study of silty soil samples has been conducted on samples prepared by the moist tamping method. However, it has been complained that the increase in the fine content leads to large volumetric changes [23, 31, 32]. It is argued that the moist tamping method does not simulate the fabric of alluvial soil deposits and does not assure specimen uniformity [33]. The moist tamping specimens of loose saturated sand containing fines are typically the most contractive [4, 34]. Uniform sand specimens can be prepared by the dry pluviation method and wet pluviation method. The dry pluviation method simulates soil of aeolian deposits. However, dry pluviation does not simulate the silty sand process or void ratios for hydraulic fills because dry pluviation includes particle segregation and soil bulking with large fine content. The wet pluviation method conducts water and ensures specimen saturation. This method simulates depositional environments such as alluvial soils. However, the problem is that particle segregation can occur with poorly graded sand [16].

The slurry deposition method was presented by Ishihara et al. [35] for silty sand and sandy silt. Kuerbis and Vaid [16] developed a new method of specimen preparation called slurry deposition that produces homogenous specimens of sand and silty sand. In this method sand and water are mixed. The saturated soil specimen is transferred to a clear plexiglass tube and maintains saturation. They preferred to prepare fine-grained samples with more than 20% fines using the slurry deposition method. Salgado et al. [36] used to prepare triaxial sand containing up to 20% silt specimens. The slurry method permits homogeneous void ratios and repeatable specimen densities.

Mulilis et al. [37] studied the influence of the sample preparation method on the liquefaction behavior of sandy soil using dry/wet pluviation, high frequency vibration, and dry/wet rodding. The results suggested that although similar relative densities were achieved their paths to liquefaction were not identical. Ladd [38] prepared samples of sand employing dry vibration, wet vibration, dry tamping, and wet tamping methods and reported that the method of sample preparation had a profound effect on the dynamic behavior of sand. Wagg [39] studied the response of clay-silt mixtures to dynamic loading. He preferred to mix the air-dried samples with sufficient water to bring the slurry to a water content of 1.5 wL. Vaid et al. [34] studied the changes in the liquefaction susceptibility of sand to varying silt contents. They claimed that moist tamping did not provide the structure of alluvial soil and that samples prepared by this method had unusually high void ratios. They preferred the slurry method which produced homogeneous samples with realistic void ratios.

Polito and Martin [40] studying the effect of nonplastic fines on liquefaction resistance reported that samples prepared by moist tamping which had higher relative density showed higher liquefaction resistance compared to the samples prepared by the slurry technique. In another study, water soil mixtures prepared in slurry form were placed in a mould and consolidated by air pressure at 150 kPa, after which tubes were pushed to obtain samples [41]. Yamamuro and Wood [42] performed cyclic triaxial tests on Nevada sand with 20% silt using different preparation methods such as dry pluviation, pouring through a funnel, sedimenting in water, and slurry deposition and found that such methods produced quite different textures, although the void ratios were the same. Accordingly, it was decided that the samples would be prepared by slurry deposition as it appeared to produce reasonably uniform samples. Moist tamping has also been widely employed to prepare specimens of granular soil and was also found to be suitable for silt samples in certain applications where the moist soil was laid on several lifts into a mould and tamped to the desired compaction [43]. In this paper, the slurry deposition method was chosen for the study as it enables producing reasonably representative silt samples. The advantages of the method are its easy and rapid sample preparation, full saturation, control of void ratio, and homogeneity.

In this paper, soil samples were prepared with the primary target of a constant void ratio. To ensure this, the dry sample was thoroughly mixed with distilled water at 1.5 times its liquid limit and left to stand for 24 hours. This mixture was then poured into a cell of 10 cm. diameter and 22 cm. height and left to stand overnight before starting the consolidation process by loading the sample in about ten increments to reach the prescribed pressure of 100 kPa. Drainage was provided at the top and bottom. The samples reached full consolidation within a week to a fortnight, depending on the clay content. The sample was then placed in a freezer overnight to allow it to gain sufficient strength to stand up during preparation for testing, before being extruded. All the samples tested in the program had void ratios between 0.75 and 0.80 to eliminate the possible effects on dynamic behavior. In this study, samples which were outside these limits were not used. The samples were placed in the triaxial cell in frozen condition and left to thaw for 24 hours. Saturation and consolidation stages were implemented despite the fact that samples had been consolidated to the desired pressures during deposition.

### 2.3. Cyclic Triaxial Test System

**Figure 2:**
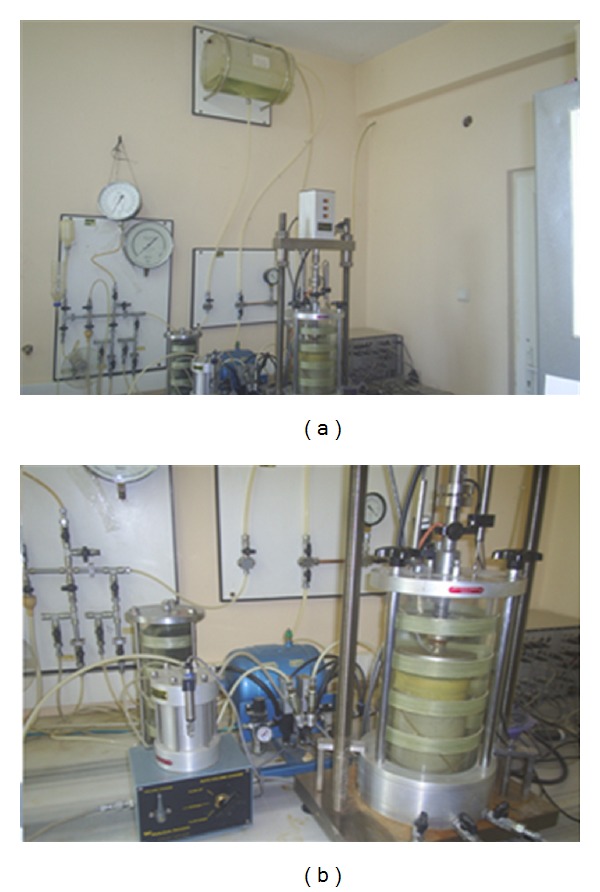
The cyclic triaxial test system.

**Figure 3:**
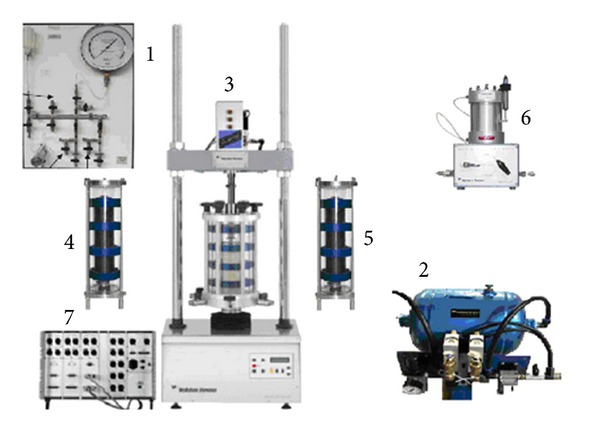
Equipment of cyclic triaxial system.

In this research, the pneumatic testing system was used to perform the stress-controlled cyclic triaxial test at Sakarya University (Figure 2). The cyclic triaxial test was conducted using “Universal Testing Machine” (UTM) software, developed by Wykeham Farrance.  Figure 3 shows the cyclic triaxial test system equipment. The parts comprise (1) water and air circulation system, (2) air dryer, (3) loading frame, (4) and (5) unchanged pressure cells, (6) measurement of changes in volume, and (7) control and data acquisition system (CDAS).

After the sample soil was placed in the cell, the cyclic triaxial test was conducted in three phases, namely, saturation, consolidation, and cyclic loading. In the saturation phase, saturation was checked according to ASTM D 5311-92 (2004). The degree of saturation control was conducted by Skempton's parameter (*B*). According to ASTM D 5311-92 [44], test samples are considered to be fully saturated if *B*-value is equal to or greater than 0.95. After the samples were fully saturated, silty soil samples were subjected to isotropic consolidation. When consolidation was complete, the silty soil was exposed to the double amplitude of sinusoidal varying dynamic loads at the appropriate cyclic stress ratio.

## 3. Cyclic Triaxial Test Results

The physical properties of the reconstituted samples with 4, 6, 9, 10, and 12 percent clay content are presented in Table 1. The stress-controlled cyclic triaxial test was used to evaluate the dynamic behavior of all silty soil. The size of the cyclic triaxial test samples is 100 mm in diameter. The testing system individually enables the measurement and the recording of axial vertical load, axial vertical displacement, pore water pressure, the specimen volume change, effective pressure, and consolidation ratio. Firstly, cyclic triaxial tests were calibrated to Monterey sand in order to compare the results with those of previous investigations [45, 46]. The Wykeham Farrance system was shown to produce results consistent with those of previous studies.

The factors that influence the dynamic behavior in the cyclic triaxial test cell have been listed as the method of sample preparation, grain size distribution, relative density, the *B* parameter, method and the frequency of loading, the initial stress ratio, and sample size [47]. Silver [48] emphasized the importance of sample size. His findings have been supported by other researchers. Freezing the sample has not been found to influence the results significantly. A preliminary study was performed to minimize those factors before cyclic triaxial testing was initiated. Nicholson and Kashyap [49] stated that membrane effect is eliminated if the samples have D20 ≤ 0.2 mm. This effect was not taken into account since the silt samples had smaller dimensions. The effect of sample size was evaluated next. Cyclic triaxial tests were performed on 50 mm and 100 mm diameter samples. In terms of the development of excess pore pressure during shear, it was shown that the excess pore pressure ratio reached *r*_*u*_ = 1 after *N* = 20 cycles for the 100 mm samples, whereas this state was never reached for the 50 mm samples and an excess pore pressure ratio of 0.8 was reached only after *N* = 50 cycles, indicating a significantly lower resistance for larger samples. Accordingly, 100 mm samples were employed for the testing program.

Sancio [45] studied the influence of frequency (*f*) by testing at 1 Hz and 0.005 Hz and showed that decreasing frequency reduced the number of cycles required to reach failure. Samples were tested at 1 Hz, 0.5 Hz, and 0.05 Hz for this study to adopt the suitable *f* value. At frequency *f* = 1 Hz, the pore pressure ratio of soil never reached *r*_*u*_ = 1, whereas at frequencies *f* = 0.5 Hz and 0.005 Hz the pore pressure ratio of soils easily reached *r*_*u*_ = 1. Therefore, it was found that *f* = 0.5 Hz was the most suitable to expose the dynamic behavior of the samples. At the end of this preliminary study, it was decided to minimize the variables for 100 mm samples reconstituted by the slurry deposition method. The initial effective consolidation stress of 100 kPa and a frequency of 0.5 Hz were used for the tests. All specimens were conducted with those values. The cyclic triaxial test was applied to all specimens with those values; thus was the effect of an effective consolidation pressure and the loading frequency removed.

The stress-controlled cyclic triaxial test was used to evaluate the dynamic behavior of all silty soil. The size of cyclic triaxial test samples was 100 mm in diameter. All the samples tested were isotropically consolidated. Cyclic stress ratios (CSR) of 0.15, 0.20, 0.25, and 0.375 were applied to the samples. Failure criteria were defined and evaluated as 5% double amplitude axial strain in 15 cycles of loading, representing an earthquake of magnitude Mw = 7.5 as recommended by Seed and Idriss [50].

**Figure 4:**
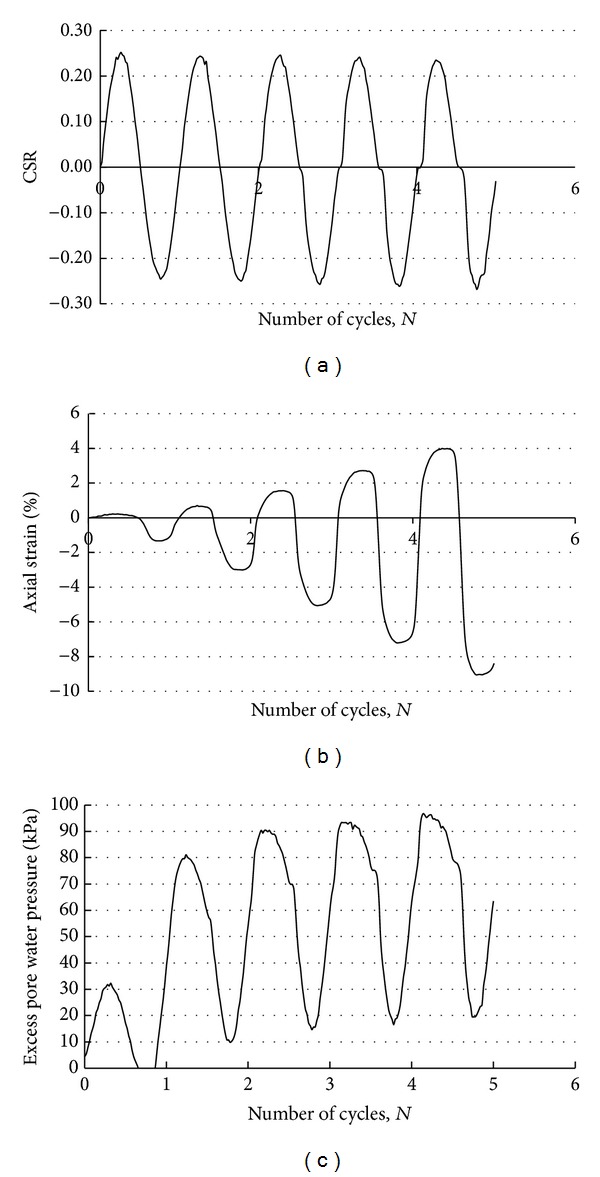
Cyclic triaxial test results of silty soil a having content 54% fine 4% clay for CSR = 0.25.

The cyclic triaxial tests were conducted at stress ratios of 0.15, 0.20, 0.25, and 0.375, respectively.  Table 2 shows the results of the cyclic triaxial test with varying cyclic stress ratios (CSR).  Figure 4 presents the results of a typical test graph. This graph shows the relationships between cyclic stress ratio and the number of cycles, axial strain and the number of cycles, and excess pore water pressure and the number of cycles of silty soil samples with a fine content of 54% and clay content of 4%.

### 3.1. The Influence of CSR on Pore Pressure Generation

**Figure 5:**
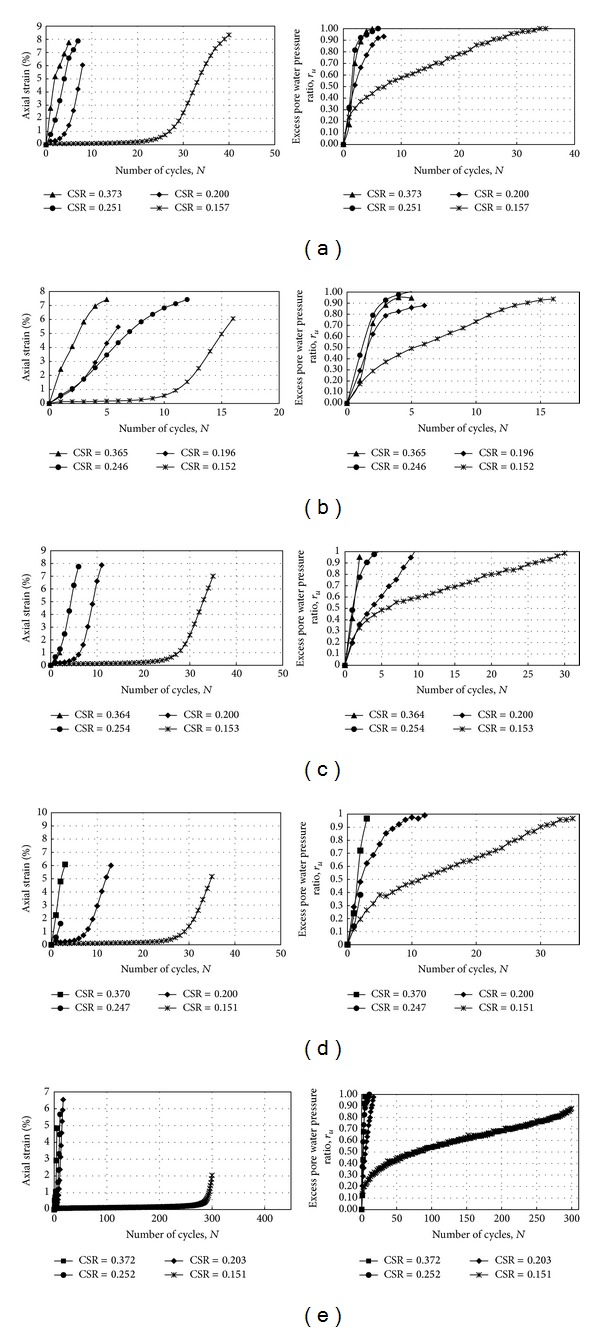
The excess pore water pressure-number of cycles relationship and the axial strain-number of cycles relationship for different grain size distributions (a) 54% fine 4% clay. (b) 51% fine 6% clay. (c) 71% fine 9% clay. (d) 67% fine 10% clay. (e) 73% fine 12% clay.

The relationship between axial strain and number of cycles and between excess pore water pressure ratio and number of cycles for soil with 54% fine content and 4% clay content can be observed in Figure 5(a). A cyclic stress ratio of 0.157 required 30 cycles for 5% double amplitude axial strain. With this number of cycles, the excess pore water pressure reached 96.22 kPa. A cyclic stress ratio of 0.200 required 6 cycles for 5% double amplitude axial strain. With this number of cycles, the excess pore water pressure reached 91.82 kPa. A cyclic stress ratio of 0.251 required 3 cycles for 5% double amplitude axial strain. With this number of cycles, the excess pore water pressure reached 91.33 kPa. A cyclic stress ratio of 0.373 required only 1 cycle for 5% double amplitude axial strain. With this number of cycles, the excess pore water pressure reached 17.09 kPa.

The relationship between axial strain and number of cycles and between excess pore water pressure ratio and number of cycles for soil with 51% fine content and 6% clay content can be observed in Figure 5(b). A cyclic stress ratio of 0.152 required 13 cycles for 5% double amplitude axial strain. With this number of cycles, the excess pore water pressure reached 88.89 kPa. A cyclic stress ratio of 0.196 required 4 cycles for 5% double amplitude axial strain. With this number of cycles, the excess pore water pressure reached 83.03 kPa. A cyclic stress ratio of 0.246 required 4 cycles for 5% double amplitude axial strain. With this number of cycles, the excess pore water pressure reached 98.17 kPa. A cyclic stress ratio of 0.365 required only 1 cycle for 5% double amplitude axial strain. With this number of cycles, the excess pore water pressure reached 20.03 kPa.

The relationship between axial strain and number of cycles and between excess pore water pressure ratio and number of cycles for soil with 71% fine content and 9% clay content can be observed in Figure 5(c). A cyclic stress ratio of 0.152 required 30 cycles for 5% double amplitude axial strain. With this number of cycles, the excess pore water pressure reached 98.17 kPa. A cyclic stress ratio of 0.199 required 8 cycles for 5% double amplitude axial strain. With this number of cycles, the excess pore water pressure reached 86.45 kPa. A cyclic stress ratio of 0.254 required 3 cycles for 5% double amplitude axial strain. With this number of cycles, the excess pore water pressure reached 87.91 kPa. A cyclic stress ratio of 0.364 required 2 cycles for 5% double amplitude axial strain. With this number of cycles, the excess pore water pressure reached 95.24 kPa.

The relationship between axial strain and number of cycles and between excess pore water pressure ratio and number of cycles for soil with 67% content and 10% clay content can be observed in Figure 5(d). A cyclic stress ratio of 0.151 required 32 cycles for 5% double amplitude axial strain. With this number of cycles, the excess pore water pressure reached 92.31 kPa. A cyclic stress ratio of 0.201 required 10 cycles for 5% double amplitude axial strain. With this number of cycles, the excess pore water pressure reached 96.70 kPa. A cyclic stress ratio of 0.247 required 3 cycles for 5% double amplitude axial strain. With this number of cycles, the excess pore water pressure reached 62.03 kPa. A cyclic stress ratio of 0.369 required 1 cycle for 5% double amplitude axial strain. With this number of cycles, the excess pore water pressure reached 23.93 kPa.

The relationship between axial strain and number of cycles and between excess pore water pressure ratio and number of cycles for soil with 73% fine content and 12% clay content can be observed in Figure 5(e). A cyclic stress ratio of 0.151 required 302 cycles for 5% double amplitude axial strain. With this number of cycles, the excess pore water pressure reached 87.42 kPa. A cyclic stress ratio of 0.203 required 12 cycles for 5% double amplitude axial strain. With this number of cycles, the excess pore water pressure reached 79.61 kPa. A cyclic stress ratio of 0.252 required 8 cycles for 5% double amplitude axial strain. With this number of cycles, the excess pore water pressure reached 94.26 kPa. A cyclic stress ratio of 0.372 required 4 cycles for 5% double amplitude axial strain. With this number of cycles, the excess pore water pressure reached 91.82 kPa. Figures 5(a)–5(e) show the relationship between pore water pressure ratio and the number of cycles and the relationship between axial strain and the number of cycles for different cyclic shear ratios. In general, for soil with clay content equal to or less than 10%, the excess pore pressure ratio buildup was quicker with an increase in CSR.

### 3.2. The Influence of Grain Size Distribution on Excess Pore Water Pressure Generation

**Figure 6:**
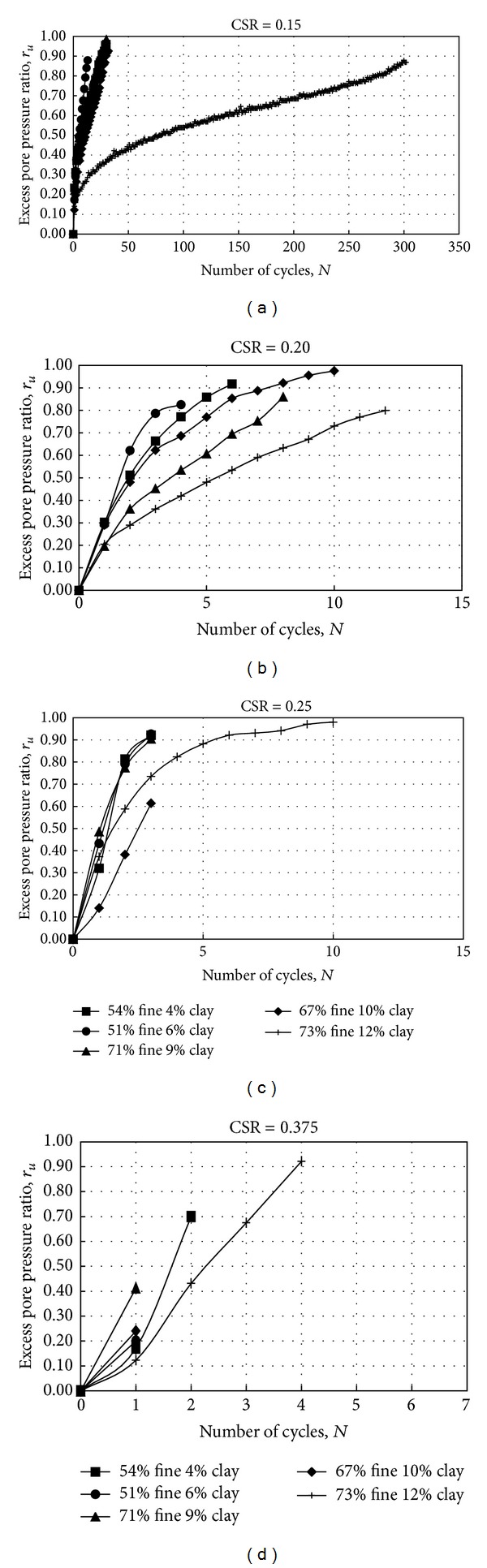
The pore pressure-number of cycles and axial strain-number of cycles relationship for a different CSR.

In Figure 6(a) it can be observed that, while the cyclic value was about 300 in soil with 73% fine content and 12% clay content, the cyclic value was up to about 30 in other soils for the required 5% double amplitude axial strain. When the cyclic shear ratio was 0.15, the excess pore water pressure ratio value was between 87 and 98 kPa in the number of cycles required for 5% double amplitude axial strain. In Figure 6(b) it can be observed that the cyclic value was less than 15 in all samples for the 5% double amplitude axial strain required. When the cyclic shear ratio was 0.20, the excess pore water pressure ratio value was between 79 and 97 kPa in the number of cycles required for 5% double strain amplitude.

In Figure 6(c) it can be observed that the cyclic value was the first 10 cycles in all samples for the 5% double strain amplitude required. When the cyclic shear ratio was 0.25, the excess pore water pressure ratio value was between 62 and 98 kPa in the number of cycles required for the 5% double amplitude axial strain. In Figure 6(d) it can be observed that the cyclic value was about the first five cycles in all samples for the 5% double amplitude axial strain required. When the cyclic shear ratio was 0.375, the excess pore water pressure ratio value was between 17 and 95 kPa in the number of cycles required for 5% double amplitude axial strain.

Figures 6(a)–6(d) show the relationship between pore water pressure ratio and the number of cycles for different grain size distributions where the cyclic stress ratio was 0.15, 0.20, 0.25, and 0.375. In general, when the fine and clay content increased, the excess pore water pressure decreased the constant cyclic shear ratio in nonplastic silty soil. During cyclic loading, for all soils, the excess pore water pressure ratio increased rapidly with an increasing number of loading cycles and increasing cyclic stress ratio. While the cyclic stress ratio increased, the pore water pressure value decreased in the number of cycles required to reach 5% double amplitude axial strain.

### 3.3. Evaluation of Cyclic Behavior of Fine-Grained Soils Considering Index Test

**Figure 7:**
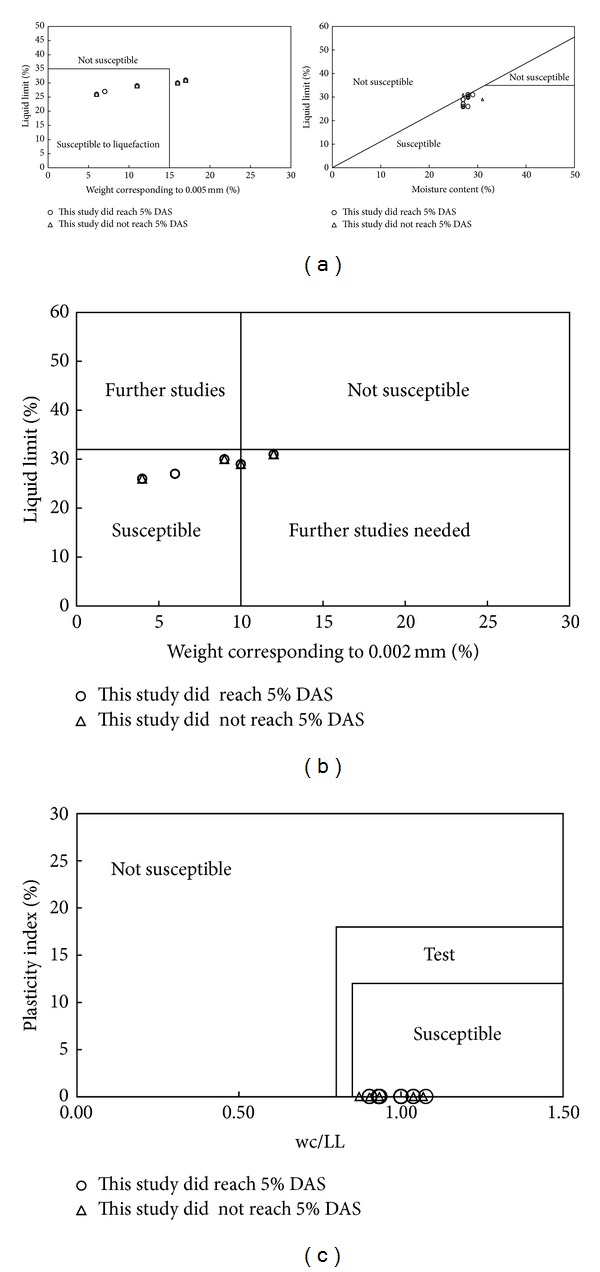
Graphical presentation of criteria. (a) The Chinese criteria [51]. (b) Andrews and Martin's criteria [52]. (c) Bray and Sancio's criteria [53].

In this section of the study the soil used in the tests was evaluated using index test data and compared with the test results and criterion-based index test results and grain size data (Chinese criteria [51]) the Andrews and Martin's [52] criteria, and the Bray and Sancio's [53] criteria). According to Figure 7, the laboratory test results indicate that the Chinese criteria [51], Andrews and Martin's [52] and Bray and Sancio's [53] criteria may not always be valid when assessing the liquefaction susceptibility of silty soil. The Chinese criteria were based on observed failures due to liquefaction in fine-grained soil after earthquakes which occurred in China [51]. It is thought that there is disagreement between Chinese criteria and laboratory results due to the fact that the Chinese criteria were based on liquefaction identified by the observable surface (Figure 7(a)).

Andrews and Martin [52] suggested that fine-grained soil has liquefaction potential if the percentage of fine content by weight smaller than 2 *μ*m is less than 10% and the liquid limit is less than 32%. They stated that when liquefiable soil has a liquid limit less than 32% clay content is less than 10%. In addition, when the liquid limit value is less than 32%, clay content is equal to or greater than 10% and when the liquid limit is equal to or greater than 32%, clay content is less than 10%, indicating a need for further testing of the area. Soil with a liquid limit equal to or greater than 32% and clay content equal to or greater than 10% will not liquefy under normal circumstances. According to Figure 7(b), the same samples were found to be susceptible to liquefaction, though these criteria suggest that they are not susceptible and further studies are needed. Consequently, it arises as suspected that these criteria cannot identify liquefaction sufficiently.

Bray and Sancio [53] proposed that fine-grained soil with a plasticity index equal to or less than 12 and natural water content equal to or greater than 0.85 liquid limit has liquefaction potential. Partial sensitivity is indicated with a plasticity index within the limits of 12 to 18 and natural water content equal to or higher than 0.80 liquid limit. Soil with a plasticity index greater than 18 and natural water content lower than a liquid limit of 0.80 will not liquefy. According to Figure 7(c), these criteria suggest that all the samples are susceptible, though they were found to be both susceptible to liquefaction and not susceptible to liquefaction. Nonplastic silty soil with a plasticity index equal to 12 is unlikely to be a realistic proposal. For this reason, the proposal was seen to be disputable.

## 4. Conclusions 

The present study focused on how the influence of the clay fraction affected dynamic properties under different cyclic stress ratios. Therefore, a series of stress-controlled isotropic and undrained cyclic triaxial tests was conducted on silty soil to examine the dynamic properties. The logic for this kind of approach was justified because Adapazarı silt contains different percentages of the same clay minerals, the level of which has been altered by the varying rates of flow of the Sakarya River. The cyclic triaxial tests were conducted on silty soil reconstituted by slurry deposition. The nonplastic samples exhibited widely varying behavior under increasing cyclic stress ratios. It was shown that in general for soil with a clay content equal to or less than 10% the excess pore pressure ratio buildup was quicker with an increase in CSR. When fine and clay content increased, the excess pore water pressure decreased the constant cyclic shear ratio in nonplastic silty soil. The Chinese criteria, Andrews and Martin's 2000 criteria, and Bray and Sancio's 2006 criteria may not be suitable for determining the liquefaction susceptibility of these silty soils because soil liquefaction is highly affected by the grain size distribution, void ratio, and, especially, the laboratory test values of the effective consolidation. Finally, known liquefaction criteria may not always be valid when assessing the dynamic behavior of nonplastic silt.

## Figures and Tables

**Table 1:** The physical properties of the silt mixtures used in testing.

Mixture	*w*_*L*_	*w*_*P*_	*I*_*P*_	*G*_*s*_	Sand (%)	Fine content (%)	Silt (%)	Clay (%)
1	27	—	NP	2.73	46	54	50	4
2	28	—	NP	2.72	49	51	45	6
3	31	—	NP	2.69	29	71	62	9
4	30	—	NP	2.70	33	67	57	10
5	32	—	NP	2.69	27	73	61	12

**Table 2:** The physical and dynamic properties of the samples.

Sample	FC (%)	*C* (%)	*w*_*n*_ (%)	*ρ* (kN/m^3^)	*σa* (kPa)	CSR	*N* for 5% DAS	Δ*U*_*w*_ for 5% DAS (kPa)
1.1	54	4	28	19.08	74.24	0.373	1	17.09
1.2	54	4	27	18.76	49.80	0.251	3	91.33
1.3	54	4	27	19.16	39.99	0.200	6	91.82
1.4	54	4	27	18.85	31.36	0.157	30	96.22
2.1	51	6	27	19.26	73.72	0.365	1	20.03
2.2	51	6	27	19.49	49.49	0.246	4	98.17
2.3	51	6	27	19.93	39.59	0.196	4	83.03
2.4	51	6	27	19.35	30.82	0.152	13	88.89
3.1	71	9	28	18.92	72.87	0.346	2	95.24
3.2	71	9	30	18.58	49.62	0.254	3	87.91
3.3	71	9	28	18.61	40.23	0.199	8	86.45
3.4	71	9	28	18.77	30.39	0.152	30	98.17
4.1	67	10	28	17.68	73.56	0.369	1	23.93
4.2	67	10	27	17.06	49.96	0.247	3	62.03
4.3	67	10	27	19.08	40.00	0.201	10	96.70
4.4	67	10	31	18.95	30.05	0.151	32	92.31
5.1	73	12	28	18.49	73.74	0.372	4	91.82
5.2	73	12	28	18.40	50.24	0.252	8	94.26
5.3	73	12	29	19.08	40.50	0.203	12	79.61
5.4	73	12	27	18.82	30.31	0.151	302	87.42

DAS: double axial strain amplitude. *N*: cycles. Δ*U*_*w*_: excess pore water pressure.
